# The soluble guanylyl cyclase activator BAY 60-2770 ameliorates detrusor dysfunction in obese mice

**DOI:** 10.1186/2050-6511-14-S1-P18

**Published:** 2013-08-29

**Authors:** Gilberto de Nucci, Luiz Osorio Leiria, Fábio Henrique da Silva, Eduardo C Alexandre, Marina Calixto, Fabíola Zakia Mónica, Edson Antunes

**Affiliations:** 1 Department of Pharmacology, State University of Campinas (UNICAMP), Brazil; 2 Institute of Biomedical Sciences, University of Sao Paulo (USP), Brazil

## Background

The obesity-associated insulin resistance has been shown to play an important role in the pathophysiology of overactive bladder in mice [1,2]. Therefore, we evaluated the beneficial effects of long-term administration of the sGC activator BAY 60-2270 in bladders from lean and obese mice.

## Methods

Mice were fed for 12 weeks with either a standard chow diet (carbohydrate: 70%; protein: 20%; fat: 10%) or a high fat diet that induces obesity (carbohydrate: 29%; protein: 16%; fat: 55%). Lean and obese mice were orally treated with BAY 60-2770 (1 mg/kg/day, given as daily gavage from the 10^th^ to the 12^th^ week) or its vehicle (Transcutol^®^:Cremophor^®^:water, 1:2:7, v/v/v). Concentration-response curves to full agonist carbachol (CCh, 0.001-100 µM) were obtained. The values of potency (pEC_50_) and maximal responses (E_max_) were calculated. The cGMP levels and Western blotting for α_1_ and β_1_-subunit of sGC in the bladder tissues were also determined.

## Results

**Figure 1:**
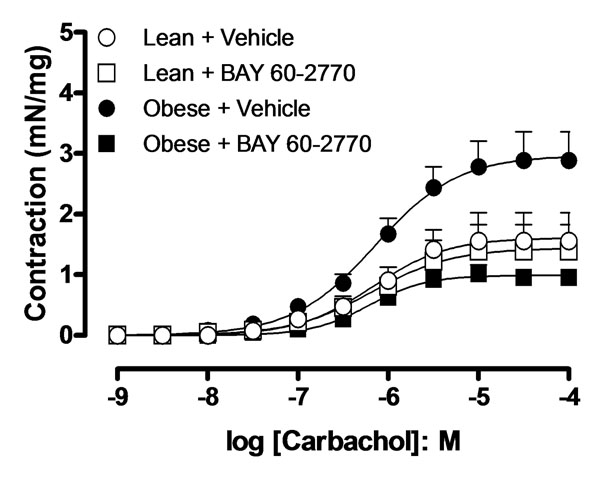
Concentration response curve to cabachol (0.001-100 µM) in isolated bladder from lean and obese mice that received or not BAY 60-2770 (1 mg/Kg, 2 weeks). Data represent mean ± S.E.M.

**Figure 2:**
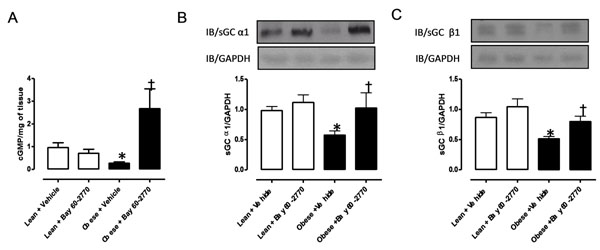
Effect of chronic treatment with BAY 60-2770 (1 mg/kg, 2 weeks) on cGMP levels (A) and protein expression of α_1_ (B) and β_1_ (C) subunits of sGC in bladders from lean + vehicle, lean + BAY 60-2770, obese + vehicle and obese + BAY 60-2770 groups. Data are presented as mean ± SEM. ★p <0.05 in comparison with lean + vehicle group; †p <0.05 in comparison to obese + vehicle group.

Contractile response to the muscarinic agonist carbachol was greater (p<0.05, n=5) in bladder from the obese in comparison with lean group. Long-term treatment with BAY 60-2770 normalized the enhanced contractile responses of the obese group, driving it to control levels (p<0.05; figure 1). The cGMP levels in the bladder tissues from obese group were significantly lower in comparison with lean mice (0.27 ± 0.04 and 0.95 ± 0.14 pmol/mg tissue, respectively, p<0.05, n=5). Treatment with BAY 60-2770 generated a 10-fold increase (p<0.01) in the bladder cGMP levels of obese mice, without affecting the levels in the lean group (Figure 2A). Protein expression of α_1_ and β_1_ subunits of sGC was decreased by 41% and 43% (p<0.05) in bladder tissues of obese animals, respectively. However, oral treatment with BAY 60-2770 restored the protein levels of α_1_ and β_1_ subunits to that of lean group (Figure 2B and 2C).

## Conclusion

Chronic treatment with BAY 60-2770 results in amelioration of bladder dysfunction in high-fat obese mice.
